# Central NPY‐Y5 sub‐receptor partially functions as a mediator of NPY‐induced hypothermia and affords thermotolerance in heat‐exposed fasted chicks

**DOI:** 10.14814/phy2.13511

**Published:** 2017-12-06

**Authors:** Hatem M. Eltahan, Mohammad A. Bahry, Hui Yang, Guofeng Han, Linh T. N. Nguyen, Hiromi Ikeda, Mohamed N. Ali, Khairy A. Amber, Mitsuhiro Furuse, Vishwajit S. Chowdhury

**Affiliations:** ^1^ Laboratory of Regulation in Metabolism and Behavior Graduate School of Bioresource and Bioenvironmental Sciences Faculty of Agriculture Kyushu University Fukuoka Japan; ^2^ Agriculture Research Center Animal Production Research Institute Agriculture Ministry Cairo Egypt; ^3^ Division for Poultry Production Faculty of Agriculture Kafr‐Elsheikh University Kafr‐Elsheikh Egypt; ^4^ Division for Experimental Natural Science Faculty of Arts and Science Graduate School of Bioresource and Bioenvironmental Science Kyushu University Fukuoka Japan

**Keywords:** Fasted chicks, HSP, NPY sub‐receptors, NPY, rectal temperature

## Abstract

Exposure of chicks to a high ambient temperature (HT) has previously been shown to increase neuropeptide Y (NPY) mRNA expression in the brain. Furthermore, it was found that NPY has anti‐stress functions in heat‐exposed fasted chicks. The aim of the study was to reveal the role of central administration of NPY on thermotolerance ability and the induction of heat‐shock protein (HSP) and NPY sub‐receptors (NPYSRs) in fasted chicks with the contribution of plasma metabolite changes. Six‐ or seven‐day‐old chicks were centrally injected with 0 or 375 pmol of NPY and exposed to either HT (35 ± 1°C) or control thermoneutral temperature (CT: 30 ± 1°C) for 60 min while fasted. NPY reduced body temperature under both CT and HT. NPY enhanced the brain mRNA expression of HSP‐70 and ‐90, as well as of NPYSRs‐Y5, ‐Y6, and ‐Y7, but not ‐Y1, ‐Y2, and ‐Y4, under CT and HT. A coinjection of an NPYSR‐Y5 antagonist (CGP71683) and NPY (375 pmol) attenuated the NPY‐induced hypothermia. Furthermore, central NPY decreased plasma glucose and triacylglycerol under CT and HT and kept plasma corticosterone and epinephrine lower under HT. NPY increased plasma taurine and anserine concentrations. In conclusion, brain NPYSR‐Y5 partially afforded protective thermotolerance in heat‐exposed fasted chicks. The NPY‐mediated reduction in plasma glucose and stress hormone levels and the increase in free amino acids in plasma further suggest that NPY might potentially play a role in minimizing heat stress in fasted chicks.

## Introduction

Neuropeptide Y (NPY), a 36‐amino acid peptide, is multifunctional (Malva et al. 2012; Reichmann and Holzer 2016). It has the orexigenic potential to increase food intake and energy homeostasis in mammals and avians (Kuenzel et al. 1987; Kuenzel and McMurtry 1988; Furuse et al. 1997; Tachibana et al. 2004, 2006; Cline and Furuse 2012). Fasting has been shown to increase the expression of NPY in the chicken hypothalamus (Boswell et al. 1999), and brain NPY has recently been found to increase with a decrease in food intake in heat‐exposed chicks (Ito et al. 2015) and broiler‐type Taiwan country chickens (Tu et al. 2016). Rapid‐growing commercial chickens have limited tolerance for high ambient temperatures (HT), and heat stress in the summer is therefore an increasing challenge globally for high‐producing commercial chickens. Chickens at all stages are susceptible to HT, including broilers of market age (Sandercock et al. 2001; Aksit et al. 2006), adult layers (Rozenboim et al. 2007; Ebeid et al. 2012) and also young chicks (Chowdhury et al. 2014; Ito et al. 2015). In young chicks, heat stress leads to an increase in body temperature (Chowdhury et al. 2012) and a decrease in food intake and body weight gain (Chowdhury et al. 2014). Recently, we found that central administration of NPY acted as a hypothermic agent in nonheat‐exposed fasted chicks and that it reduced plasma corticosterone in fasted, but not fed, chicks that had been exposed to heat, as well as in similarly fasted but not fed chicks that were nonheat exposed (Bahry et al. 2017). Moreover, central NPY stimulated food intake in chicks at control thermoneutral temperature (CT) and HT (Bahry et al. 2017). From these findings, it is clear that the regulation of food intake and also the regulation of stress and body temperature are important functions of NPY in fasted chicks under heat stress. However, whether NPY has any protective thermotolerance action in fasted chicks under heat stress is not yet known. We hypothesized that NPY may afford thermotolerance in chicks.

Heat shock proteins (HSPs) are proteins that are evolutionarily conserved and that have important functions in the environmental adaptation of all living organisms. They protect against a variety of stressful stimuli and are important in the acquisition of thermotolerance and stress resistance (Feder and Hofmann 1999; Nollen and Morimoto 2002; Fasulo et al. 2010). They have a chaperone function under stressful conditions as well as during the de novo synthesis of polypeptides, and are also involved in a range of specific cellular processes, including protein metabolism and homeostasis, signal transduction, DNA replication, immune defense reactions and metabolic detoxification (Pockley 2003; Richter et al. 2010; Zhao and Jones 2012). HSP‐70 and HSP‐90 are the most conserved and most widely studied of all HSPs. In birds, increased expression of HSP‐70 during heat stress has been found to prevent abnormal protein synthesis and aggregation (Quinteiro‐Filho et al. 2010; Najafi et al. 2015). HSP‐90 has been shown to be the most abundant constitutively expressed protein inside the cell (Stetler et al. 2010) and to have a regulatory function for the glucocorticoid receptor (GR; Hao and Gu 2014). Hence, it is important to know whether NPY regulates HSP expression.

NPY appears to carry out its biological actions via one of its sub‐receptors or via a combination of them. The NPY sub‐receptors (NPYSRs) couple to G‐proteins (Persaud and Bewick 2014), which, in chickens, include six identified NPYSRs (Bromée et al. 2006). On the basis of their amino acid sequence, the NPYSRs have been subdivided into three subfamilies: the NPYSR‐Y1 subfamily (Y1, Y4, and Y6); the ‐Y2 subfamily (Y2 and Y7); and the ‐Y5 subfamily (Y5) (Larsson et al. 2008; He et al. 2016; Gao et al. 2017). All of these NPYSRs appear to be expressed in the chicken hypothalamus, although NPYSR‐Y3 has not yet been identified in chickens (Yi et al. 2015). NPYSRs‐Y1 and ‐Y5 mediate NPY‐dependent food intake regulation in chicks (Tachibana et al. 2006; Denbow and Cline 2015). NPYSRs‐Y6 and ‐Y7 are widely expressed in all chicken tissues, but their function in the chicken genome is still unknown (He et al. 2016; Gao et al. 2017). Using NPYSRs‐Y1 and ‐Y5 antagonists, Dark and Pelz (2008) demonstrated that NPYSR‐Y1 was involved in hypothermia in cold‐acclimated Siberian hamsters. Therefore, the NPY antagonist is useful for revealing the functions of NPY‐mediated hypothermia. To the best of our knowledge, there are no reports concerning the effect of NPY on the expression of NPYSRs or on receptor functions in connection with thermoregulation in chickens.

Heat stress has been found to increase plasma corticosterone (Ito et al. 2015) and glucose (Chowdhury et al. 2012) concentrations in chicks. Central administration of NPY has been shown to decrease plasma glucose concentrations in fasted chicks under CT (Tachibana et al. 2006; Bahry et al. 2017) and to decrease plasma triacylglycerol and corticosterone concentrations in heat‐exposed fasted chicks (Bahry et al. 2017). The concentration of several plasma amino acids was found to alter as a result of short‐term (Ito et al. 2014) and long‐term heat stress (Chowdhury et al. 2014) in chicks. Recently, D‐aspartate (Erwan et al. 2014) and L‐citrulline (Chowdhury et al. 2017) have been found to play important roles in regulating body temperature and affording thermotolerance in chicks. An examination of the effects of NPY on changes in plasma metabolites, stress hormones, and amino acids in heat‐exposed chicks is therefore also needed.

In this study, we aimed to investigate the function of NPY and its NPYSR‐Y5 antagonist in relation to body temperature regulation in fasted chicks. We further aimed to determine brain expression of HSPs and NPYSRs, plasma metabolites and free amino acids, as well as stress hormones, corticosterone, norepinephrine (NE), and epinephrine (E) to reveal the role of NPY in heat‐exposed fasted chicks.

## Materials and Methods

### Animals

One‐day‐old male layer chicks (Julia strain; *Gallus gallus domesticus*) were obtained from a local hatchery (Murata Hatchery, Fukuoka, Japan) and kept under a constant room temperature of 30 ± 1°C and in continuous light in metal wire‐meshed cages (50 × 35 × 33 cm) in groups of 20–25 birds until they were 4 or 5 days old. Food (Adjust diets (metabolizable energy: >12.55 MJ/kg, protein: >23%)); Toyohashi Feed and Mills Co. Ltd., Aichi, Japan) and water were provided ad libitum. This study was conducted in accordance with the guidelines for animal experiments in the Faculty of Agriculture of Kyushu University and with Law No. 105 and Notification No. 6 of the Japanese government.

### Drug preparation and intracerebroventricular (i.c.v.) injection

NPY (Porcine, Peptide Institute, Osaka, Japan) and CGP71683 (TOCRIS bioscience, Wako Pure Chemical Industries, Ltd, Bristol, U.K.), an NPYSR‐Y5 antagonist (Holmberg et al. 2002), were dissolved in a vehicle of 0.85% saline containing 0.1% Evans Blue (Wako Pure Chemical Industries, Ltd., Osaka, Japan). Porcine NPY showed a similar affinity to chicken NPYSRs‐Y1, ‐Y4, and ‐Y5 (Lundell, 2002), so we used it in this study. Evans Blue saline solution was used for the control group, as in previous studies (Tachibana et al. 2006, 2007; Bahry et al. 2017). The NPY, the NPYSR‐Y5 antagonist and the saline solutions were kept on ice during the experimental period. I.c.v. injections were performed following the method of Davis et al. (1979). Briefly, the head of the chick was inserted into an acrylic device which positioned a hole in a plate overlying the skull immediately over the left lateral ventricle. A micro syringe was then inserted into the left lateral ventricle through the hole and the drug was injected. The syringe was kept in place for 10 s to prevent overflow. Chicks were not anesthetized, as in previous studies the i.c.v. injection of 0.85% saline, which was used for control injections, was not found to affect feeding behavior (Furuse et al. 1999) or plasma corticosterone concentrations (Saito et al. 2005) compared with noninjected chicks. Chicks in this study moved normally immediately after the injection. At the end of the experiment, following euthanasia, the brain was sliced and the presence of Evans Blue dye in the lateral ventricle was confirmed. The results from chicks without Evans Blue dye in the lateral ventricle were not used for further analysis.

### Experimental design

In Experiment 1, chicks (6 and 7 days old) were isolated in individual plastic cages (floor space: 15 cm × 28 cm; height: 13 cm) for 48 h prior to the start of the experiment for adaptation. On the day of the experiment, chicks (*n* = 16) were intracerebroventricularly injected with 10 *μ*L of either 0 or 375 pmol NPY based on our previous reports showing that 375 pmol was the effective dose of NPY (Tachibana et al. 2006; Bahry et al. 2017). Chicks were returned to their cages and placed in a temperature‐controlled chamber (Sanyo Electric Co. Ltd., Japan; catalog number: Sanyo MIR‐253) under fasting condition and maintained at either HT (35 ± 1°C) for the heat stress challenge or CT (30 ± 1°C) for 1 h. We chose the HT (35 ± 1°C) based on our previous report where the thermoneutral temperature was 30 ± 1°C (Bahry et al. 2017). The chicks’ rectal temperature was measured immediately before i.c.v. injection, and this was considered the data at 0 min, followed by measurements at 30 and 60 min after the treatment. Rectal temperature was measured using a digital thermometer with an accuracy of ±0.1°C (Thermalert TH‐5, Physitemp Instruments Inc.); the thermistor probe was inserted into the colon (rectum) through the cloaca to a depth of 2 cm as reported previously (Chowdhury et al. 2015; Ito et al. 2015; Bahry et al. 2017). At the end of experiment, the chicks were euthanized following anesthesia by isoflurane (Mylan Inc., Tokyo, Japan) in order to collect blood and brain samples. Blood from the jugular vein was immediately collected into heparinized tubes and centrifuged at 10,000*g* for 4 min at 4°C to collect plasma. The collected plasma samples were stored at −80°C for further analysis of metabolites, free amino acids, corticosterone, NE, and E. The brains were dissected and the diencephalon (consisting of the thalamus and hypothalamus) was collected as described elsewhere (Kuenzel and Masson 1988; Chowdhury et al. 2014). Brain samples were snap frozen in liquid nitrogen and stored at −80°C for analysis of brain expression of HSP‐70 and ‐90 as well as of NPYSRs. Because some chick brains did not have Evans Blue dye in the lateral ventricle, the number of samples to obtain data on rectal temperature became smaller (*n* = 12–15). Then, after analysis of brain and plasma samples, the outliers were rejected by Thompson rejection test as described later in the Statistical Analysis section. Therefore, the final numbers to get the data on gene expressions and plasma metabolites became smaller (*n* = 10–14/group). We randomly chose 10 samples from each group (*n* = 10/group) for corticosterone ELISA analysis since the analysis process was sensitive to get reliable data with a minimum sample number. However, the final number became smaller (*n* = 6–7/group) to get the corticosterone data after the rejection of the outliers by Thompson rejection test.

In Experiment 2, chicks (6 days old) were isolated as described in Experiment 1. On the day of the experiment, chicks (*n* = 12) were intracerebroventricularly injected with saline (control), 375 pmol NPY alone or 375 pmol NPY plus 3750 pmol CGP71683, an NPYSR‐Y5 antagonist, under fasting conditions. The CGP71683 dose was decided upon on the basis of previous reports (Holmberg et al. 2002; Tachibana et al. 2006). Rectal temperatures were measured as described above under CT. The number of samples to determine the changes in rectal temperature finally became smaller (*n* = 8–10) due to excluding the chicks whose brains were not stained properly as explained above.

### Isolation of total RNA and quantitative real‐time PCR

Total RNA was extracted from the chick diencephalon using RNAiso Plus (TakaRa Bio Inc., Shiga, Japan), according to the manufacturer's instructions. cDNA was synthesized using 1 *μ*g of total RNA and the PrimeScript^®^ RT reagent Kit with gDNA Eraser (Takara, Shiga, Japan), according to the manufacturer's instructions. All primers were tested by carrying out routine PCR and gel electrophoresis prior to real‐time PCR (TaKaRa PCR Thermal Cycler Dices, Takara, Shiga, Japan). The cDNA of the diencephalic tissues was analyzed for expression of HSP‐70 and ‐90, as well as of NPYSRs‐Y1, ‐Y2, ‐Y4, ‐Y5, ‐Y6, and ‐Y7, by routine PCR. To quantify the expression of HSPs and NPYSRs in the diencephalon, real‐time quantitative PCR was conducted using Startagene MX 3000P (Agilent Technologies, Tokyo, Japan) with a denaturation step at 95°C for 30 sec, with 40 cycles of amplification at 95°C for 5 sec, and a primer‐specific temperature for 30 sec. The primer sequences are listed in Table 1. Relative mRNA expressions have been calculated by comparing the number of thermal cycles that were needed to generate threshold amounts of product (PCR‐ct). PCR‐ct was calculated for the HSPs and NPYSRs and for the chicken RNA polymerase‐II (RP‐II). It was confirmed that the RP‐II expression level was not altered under the current experimental conditions. For each cDNA sample, the PCR‐ct for RP‐II was subtracted from the PCR‐ct for HSP‐70, ‐90, NPYSRs‐Y1, ‐Y2, ‐Y4, ‐Y5, ‐Y6, ‐Y7, and GR to give the parameter ΔPCR‐ct, thus normalizing the initial amount of RNA used. The HSP, NPYSR, and GR mRNA expression was calculated as 2^−ΔΔPCR‐ct^, where ΔΔPCR‐ct is the difference between the ΔPCR‐ct of the two cDNA samples to be compared, as described elsewhere (Schmittgen and Livak 2008). The single melting peak for each sample was detected to confirm the specificity of the PCR conditions.

**Table 1:** Primers used for real‐time PCR

Gene	Accession no.	Sequences 5′−3′ (forward/reverse)	Annealing temperature (°C)	Product size (bp)
*HSP‐70*	AY143691.1	5′−GGGAGGACTTTGACAACCGA−3′/5′−CAAAGCGTGCACGAGTGATG−3′	60	219
*HSP‐90*	NM_001109785	5′−GAAGACTCCCAGAACCGCAA−3′/5′−ACCTGGTCCTTTGTCTCACC−3′	60	155
*NPYR‐Y1*	NM_001031535.1	5′−TAGCCATGTCCACCATGCA−3′/5′−GGGCTTGCCTGCTTTAGAGA−3′	60	58
*NPYR‐Y2*	NM_001031128.1	5′−TGCCTACACCCGCATATGG−3′/5′−GTTCCCTGCCCCAGGACTA−3′	60	58
*NPYR‐Y4*	NM_001031555.1	5′−CCTGCCCTTTCTGACCACAT−3′/5′−GGGATGCAGTATTGCAGAAGC−3′	60	165
*NPYR‐Y5*	NM_001031130.1	5′−TGATCGGTGGATGTTTGGCA−3′/5′−AGCCAACGGCCCAAATGATA−3′	60	191
*NPYR‐Y6*	NM_001044687.1	5′−GAACGAGAGCAGGTTGAGTGA−3/5′−ACGAGGTGGCACAGTGTAAA−3′	60	174
*NPYR‐Y7*	NM_001037824.1	5′−TGGCCATCTTCAGAGAGTTCC−3/5′−GGACTGACGTGGTTTTTCAGC−3′	64	208
*GR*	NM_001037826.1	5′−TATGACAGCACGCTGCCCGA−3/5′−CTACCACTTGCCGTCCTCCTAACAT−3′	62	76
*RP‐II*	NM_001006448.1	5′−CGACGGTTTGATTGCACCTG−3′/5′−CAATGCCAGTCTCGCTAGTTC−3′	64	161

Primers were designed with Primer‐Blast (http://www.ncbi.nlm.nih.gov/tools/ primer‐blast/) for heat shock protein (HSP‐70 and ‐90), neuropeptide Y receptors (NPYR‐Y1, ‐Y2, ‐Y4, ‐Y5, ‐Y6, and ‐Y7), glucocorticoid receptor (GR), and RNA polymerase‐II (RP‐II).

### Analysis of plasma corticosterone, NE, E, and metabolites

Plasma corticosterone concentrations were determined using an enzyme immunoassay kit (Corticosterone ELISA Kit, Enzo Life Science Inc., Farmingdale, NY) and expressed as ng/mL. Each plasma sample was thawed and diluted with an assay buffer by a factor of 3. Two doses of corticosterone solution in an assay buffer which gave around 80% and 20% binding on the standard curve were used as low‐ and high‐quality controls in every assay. Standards, samples and quality controls were assayed in duplicate wells. The intra‐assay coefficients of variation were 3.4% and 9.3%, and the interassay coefficients of variation were 19.8% and 27.8% for low‐ and high‐quality controls. Plasma E and NE concentrations were measured using high performance liquid chromatography (HPLC) following the method described by Takahashi et al. (2005). Fifty *μ*L plasma was diluted with 500 *μ*L Tris buffer (pH 8.6) and 100 *μ*L EDTA·2Na, and 5 mg alumina was added and shaken by a thermomixer compact covered by aluminum foil to protect it from the light, at 4°C, 90 *g*/10 min, to absorb E and NE from the plasma. After removal of the liquid, the alumina was rinsed with ultrapure water and transferred to centrifuge‐filtration units (0.22 *μ*m Ultra Free‐MC, Millipore, Massachusetts). Alumina‐absorbed E and NE were separated by adding 2% acetic acid solution containing 100 *μ*mol/L EDTA·2Na, and then the solution was filtrated at 2000*g* for 5 min at 4°C. Thirty *μ*L of filtrate was injected into an HPLC system. The mobile phase consisted of 0.1 mol/L pH 5.7 phosphoric acid buffer (2 L of 0.1 mol/L sodium dihydrogen‐phosphate and 170 mL of 0.1 mol/L disodium hydrogen‐phosphate), 296 mL methanol, 1.48 g sodium 1‐octane sulfonate (600 mg/L), and 0.12 g disodium ethylene‐diamine‐tetraacetic acid (50 mg/L) at a flow rate of 0.5 mL/min. A standard solution was prepared by diluting it with 0.01 N HCl (standard concentrations were 3000, 1500, 750, 375, 187.5, and 93.75 pg/30 *μ*L). The concentrations of NE and E in plasma were expressed as pg/*μ*L. Plasma metabolites (glucose, triacylglycerol, calcium, sodium, potassium and chloride) were determined by Dri‐Chem 7000 system (Fuji Medical System Co. Ltd., Japan).

### Analysis of free amino acids in the plasma

To analyze the effect of NPY on amino acid metabolisms, free amino acid and dipeptides were analyzed in plasma using HPLC according to the method of Boogers et al. (2008) with slight modifications as described by Ito et al. (2015). Briefly, plasma was deproteinized by filtration through a 10,000 dalton molecular weight cut‐off filter (Millipore, Bedford, MA) via centrifugation at 12,000*g* for 10 min at 4°C (MX‐307, Tommy, Japan). Each 10 *μ*L sample of the plasma was dried under reduced pressure at −100 kPa (Centrifugal Vaporizer, CVE‐200D, Eyela, Japan). The dried residues were dissolved in 10 *μ*L of 1 mol/L sodium acetate‐methanol‐triethylamine (2:2:1), re‐dried under reduced pressure and then converted to their phenylthiocarbamoyl derivatives by dissolving them in 20 *μ*L of methanol‐distilled water‐triethylamine‐phenylisothiocyanate (7:1:1:1) and allowing them to react for 20 min at room temperature. The samples were dried again and dissolved in 200 *μ*L of Pico‐Tag Diluent (Waters, Milford, CT). These diluted samples were filtrated through a 0.20 *μ*m filter (Millipore). The same methods were performed on standard solutions which were prepared by diluting a commercially available L‐amino acid solution (type ANII, type B, L‐asparagine, L‐glutamine, and L‐tryptophan; Wako, Osaka, Japan) with distilled water. The solution containing the derivatives was applied to a Waters HPLC system (Pico‐Tag free amino acid analysis column (3.9 mm × 300 mm), Alliance 2690 separation module, 2487 dual‐wavelength UV detector and Millennium 32 Chromatography manager; Waters). They were equilibrated with buffer A (70 mmol/L sodium acetate adjusted to pH 6.45 with 10% acetic acid‐acetonitrile, ratio 975:25) and eluted with a linear gradient of buffer B (water‐acetonitrile‐methanol (40:45:15) (0%, 3%, 6%, 9%, 40%, and 100%)) at a flow rate of 1 mL/min at 46°C. The concentrations of free amino acids and dipeptides (phosphoserine, aspartic acid, glutamine, *α*‐aminoadipic acid, hydroxyproline, serine, asparagine, glycine, glutamic acid, *β*‐alanine, taurine, histidine, GABA, threonine, alanine, carnosine, arginine, proline, 1 methylhistidine, anserine, 3‐methylhistidine, tyrosine, valine, methionine, cystathionine, isoleucine, leucine, phenylalanine, tryptophan, ornithine, and lysine) were determined by their absorbance at a wave length of 254 nm. The plasma amino acid concentrations were expressed as pmol/*μ*L.

### Statistics

The data relating to rectal temperature from Experiment 1 were statistically analyzed by three‐way analysis of variance (ANOVA), and the rectal temperature data in Experiment 2 were statistically analyzed by two‐way ANOVA, followed by the Tukey‐Kramer test as a post hoc analysis when a significant interaction was detected. The data concerning the expression of HSPs, NPYSRs, plasma metabolites, corticosterone, E, and NE were analyzed by two‐way ANOVA with respect to HT and NPY, and Fisher least significant different (LSD) was used as a post hoc analysis. Values were presented as means ± SEM. Statistical analysis was performed using a commercially available package – Stat View (version 5, SAS Institute, Cary 1998). All data were subjected to a Thompson's rejection test, as described by Kobayashi and Pillai (Kobayashi and Pillai 2013), to eliminate outliers (*P *<* *0.01), and the remaining data were used for the analysis among groups.

## Results

### Rectal temperature and diencephalic mRNA abundance of HSPs and GRs in chicks under CT and HT

**Figure 1:**
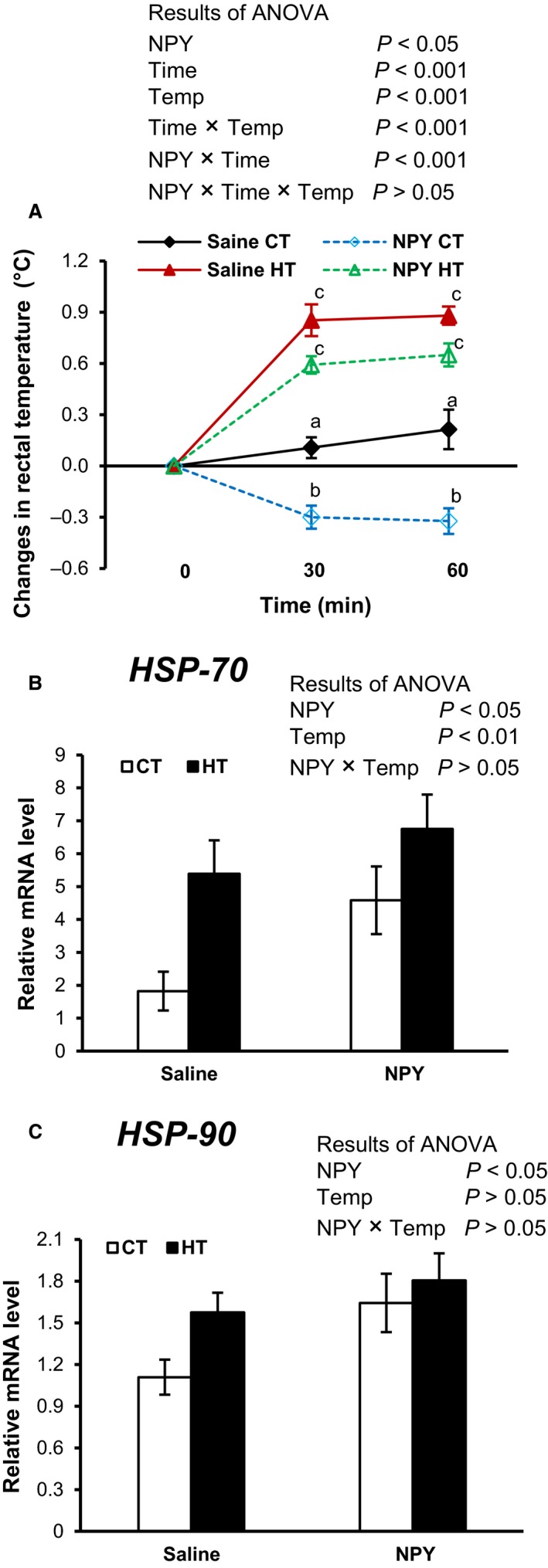
Rectal temperatures (A) and mRNA expression of diencephalic HSP‐70 (B) and HSP‐90 (C) in fasted chicks following i.c.v. injection of NPY (375 pmol) or saline under control thermoneutral temperature (CT: 30 ± 1°C) or a high ambient temperature (HT: 35 ± 1°C) for 1 h. Different letters indicate significant differences at *P *<* *0.05 between groups. Values are mean ± SEM of the 12–15 chicks in each group.

NPY i.c.v. injection significantly (*P *<* *0.05) decreased rectal temperature in fasted chicks under both CT and HT as shown in Figure 1A. There were significant (*P *<* *0.001) effects of temperature and time on changes in the rectal temperature. A significant (*P *<* *0.001) interaction was also found between NPY and time, indicating that NPY‐dependent reduction in body temperature became pronounced with special reference to CT as time went on. The expression of HSP‐70 mRNA increased significantly (*P *<* *0.05) in the brain by HT (Fig. 1B). Interestingly, NPY induced a significant (*P *<* *0.05) increment in HSP‐70 and ‐90 under both CT and HT (Fig. 1B and C). The mRNA expression of GR was not significantly changed by NPY under CT or HT (Table 3). In summary, NPY central injection decreased rectal temperature and increased brain mRNA expression of HSP‐70 and ‐90 in fasted chicks under both CT and HT.

### The diencephalic mRNA abundance of NPYSRs in chicks under CT and HT and changes in rectal temperature by the coinjection of CGP71683 (an NPYSR‐Y5 antagonist) plus NPY in chicks under CT

**Figure 2:**
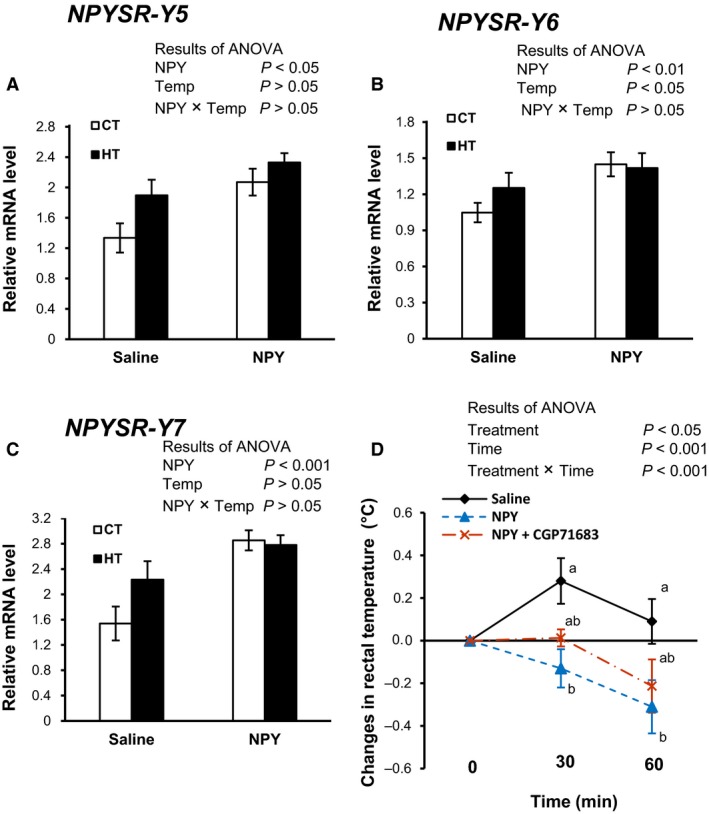
The diencephalic mRNA expression of NPYSR‐Y5 (A), NPYSR‐Y6 (B), and NPYSR‐Y7 (C) in fasted chicks following i.c.v. injection of NPY (375 pmol) or saline under control thermoneutral temperature (CT: 30 ± 1°C) or a high ambient temperature (HT: 35 ± 1°C) for 1 h. Rectal temperatures (D) of chicks following i.c.v. injection of NPY (375 pmol), saline, or NPY (375 pmol) plus CGP71683 (3750 pmol) under CT for 1 h values are mean ± SEM for each group of 10–14 chicks in A–C and 8–10 chicks in D. Different letters indicate significant differences at *P *<* *0.05 between groups.

The mRNA expression of NPYSRs‐Y5, ‐Y6, and ‐Y7 significantly (*P *<* *0.05) increased in the brain following NPY injection under both CT and HT (Fig. 2A–C). HT also significantly (*P *<* *0.05) increased NPYSR‐Y6 mRNA expression (Fig. 2B). Figure 2D shows the effect of i.c.v. NPY and coinjection of CGP71683 plus NPY on rectal temperature under CT. The NPY‐induced decreased rectal temperature was significantly (*P *<* *0.05) attenuated by coinjection of CGP71683 plus NPY. Significance of time (*P *<* *0.001) and interaction (*P *<* *0.001) between treatment and time were found, indicating that the NPY‐dependent reduction in body temperate became pronounced with the progression of time; however, CGP71683 somewhat attenuated this effect. The mRNA expression of NPYSRs‐Y1, ‐Y2, and ‐Y4 was not significantly changed by either HT or NPY (Table 3). In sum, NPY central injection caused to increase NPYSRs‐Y5, ‐Y6, and ‐Y7 significantly (*P *<* *0.05) under CT and HT. NPY‐dependent reduced rectal temperature was attenuated by the coinjection of NPYSRs‐Y5 antagonist.

### Plasma metabolites, corticosterone, NE, and E in chicks under CT and HT

**Figure 3:**
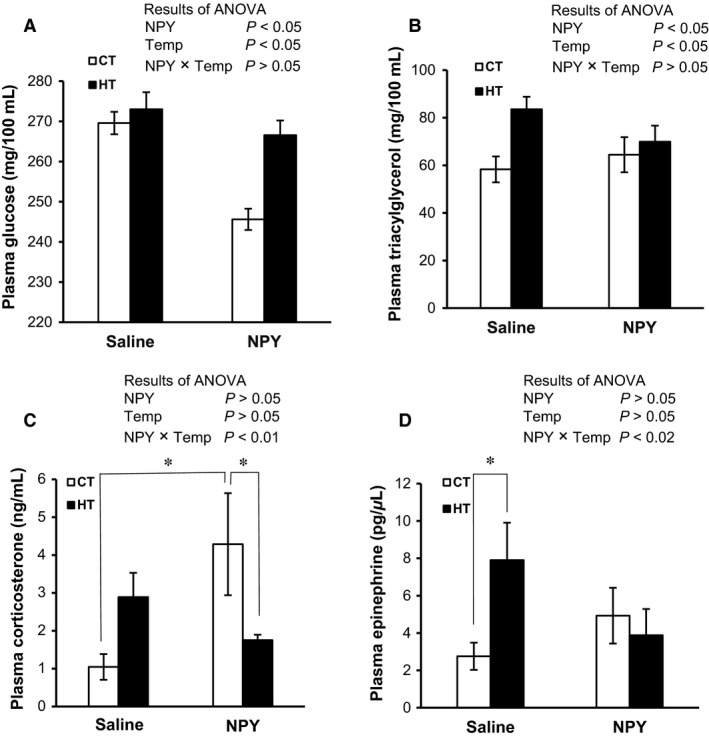
Plasma glucose (A), triacylglycerol (B), corticosterone (C), and E (D) concentrations in fasted chicks following i.c.v. injection of NPY (375 pmol) or saline under control thermoneutral temperature (CT: 30 ± 1°C) or a high ambient temperature (HT: 35 ± 1°C) for 1 h. Values are mean ± SEM for each group of 10–14 chicks in A and B and 6–7 chicks in C and D. *Significant difference between groups at *P *<* *0.05.

Plasma glucose concentrations were significantly (*P *<* *0.05) reduced by NPY i.c.v. injection (Fig. 3A). Temperature also had a significant (*P *<* *0.05) effect on plasma glucose. Although plasma triacylglycerol concentrations significantly (*P *<* *0.05) increased as a result of HT, NPY injection caused them to decrease (Fig. 3B). A significant (*P *<* *0.05) interaction between NPY and temperature was found for plasma corticosterone concentrations, indicating that they were increased in NPY‐treated chicks under CT, while this increment disappeared under HT (Fig. 3C). Moreover, a significant (*P *<* *0.05) interaction between NPY and temperature was also found for plasma E, indicating that plasma E increased in control chicks under HT; however, this stress response disappeared in NPY‐treated chicks (Fig. 3D). Plasma concentrations of calcium, sodium, potassium, chloride, and NE were not changed by NPY or HT (*data not shown*). In sum, NPY central injection reduced plasma glucose, triacylglycerol, and plasma E concentrations under CT and HT.

### Free amino acid concentrations in plasma in chicks under CT and HT

Plasma taurine and anserine significantly (*P *<* *0.05) increased following NPY i.c.v. injection in chicks under CT and HT (Table 2). Although tyrosine and valine were significantly (*P *<* *0.05) higher, histidine was lower (*P *<* *0.05) in chick plasma under heat stress. A significant (*P *<* *0.05) interaction was found between NPY and temperature for plasma valine, suggesting that the level of plasma valine was high under HT in control chicks; however, NPY caused a reduction in this level in heat‐exposed chicks (Table 2). In sum, NPY central injection increased plasma taurine and anserine under CT and HT, while heat stress increased plasma tyrosine and valine and decreased plasma histidine.

**Table 2:** Effect of high ambient temperature (35 ± 1°C, 1 h) and i.c.v. injection of NPY (375 pmol/10 *μ*L/chick) or saline on plasma‐free amino acids in fasted chicks

Amino acids	Saline		NPY		*P*‐value		
	CT	HT	CT	HT	NPY	Temperature	Temperature × NPY
Essential amino acids^a^							
Histidine	136 ± 8	107 ± 7	138 ± 6	122 ± 6	NS	*P *<* *0.005	NS
Arginine	235 ± 17	210 ± 28	238 ± 28	240 ± 23	NS	NS	NS
Leucine	271 ± 25	259 ± 50	262 ± 35	3388 ± 14	NS	NS	NS
Phenylalanine	180 ± 18	191 ± 18	168 ± 11	156 ± 10	NS	NS	NS
Threonine	443 ± 29	521 ± 99	572 ± 80	524 ± 54	NS	NS	NS
Valine	287 ± 17^a^	381 ± 21^b^	329 ± 15^ac^	332 ± 119^abc^	NS	*P *<* *0.05	*P *<* *0.05
Isoleucine	110 ± 11	143 ± 11	134 ± 12	122 ± 9	NS	NS	NS
Methionine	77.2 ± 3.5	89.0 ± 6.1	84.9 ± 4.5	80.9 ± 3.3	NS	NS	NS
Tryptophan	143 ± 10	140 ± 10	137 ± 11	146 ± 5	NS	NS	NS
Lysine	552 ± 67	610 ± 79	429 ± 45	597 ± 82	NS	NS	NS
Nonessential amino acids and dipeptide^a^							
Anserine	25.1 ± 1.9	21.1 ± 1.2	27.6 ± 2.0	26.1 ± 1.5	*P *<* *0.05	NS	NS
Taurine	43.6 ± 2.4	43.4 ± 3.9	54.5 ± 6.0	52.7 ± 4.6	*P *<* *0.0	NS	NS
Tyrosine	111 ± 8	134 ± 8	105 ± 5	117 ± 10	NS	*P *<* *0.05	NS

The number of chicks used in each group was 8. Different superscripts in the same row indicate significant differences between groups. Values are means ± SEM in pmol/*μ*L. NPY, neuropeptide Y; CT, control thermoneutral treatment (30 ± 1°C); HT, high temperature treatment; NS, not significant.

a All the analyzed essential amino acids and only significantly changed nonessential amino acids are shown.

## Discussion

In this study, we conducted i.c.v. injection of NPY in chicks to examine, in Experiment 1, its effect on thermoregulation, plasma metabolites, the stress hormone, and free amino acids in plasma, as well as the mRNA expression of HSPs and NPYSRs in the brain. In Experiment 2, an NPYSR‐Y5 antagonist was used to confirm the involvement of NPYSR in the process of NPY‐induced hypothermia.

It has been reported that body temperature reduced as a result of NPY in neonatal fasted chicks (Tachibana et al. 2006; Bahry et al. 2017) and mammals (Szekely et al. 2004; Dark and Pelz 2008) under CT. In this study, we found that central administration of NPY decreased rectal temperature not only under CT but also under HT in fasted chicks (Fig. 1A). To the best of our knowledge, this is the first report showing that NPY can afford thermotolerance. It is well known that HSP expression occurs to protect and facilitate cellular functions under thermal stress (Feder and Hofmann 1999; Nollen and Morimoto 2002; Fasulo et al. 2010; Najafi et al. 2015). It has been further reported that the expression of HSPs could be increased by the ingestion of some nutritional supplements which help to protect cellular functions against stress (Chen et al. 2012). On the other hand, HSP‐90 not only provides protective functions (Miyata and Yahara 1992; Jakob et al. 1995), it also controls the GR functions (Hao and Gu 2014). In this study, NPY showed a tendency (*P *=* *0.07) to increase GR mRNA expression (Table 3), which indicates that the NPY‐dependent changes in plasma corticosterone in this study may have some connection to HSP‐90 since HSP‐90 regulates the functions of GR (Hao and Gu 2014). NPY‐dependent expression of HSPs‐70 and ‐90 in the diencephalon implies that NPY may support protective thermotolerance and induce cellular processes in the brain under heat stress.

**Table 3:** Effect of high ambient temperature (35 ± 1°C, 1 h) and i.c.v. injection of NPY (375 pmol/10 *μ*L/chick) or saline on the diencephalic mRNA expression of NPYSRs and GR in fasted chicks

Genes	Saline		NPY		*P*‐value		
	CT	HT	CT	HT	NPY	Temperature	Temperature × NPY
NPYSR‐Y1	0.98 ± 0.53	1.14 ± 0.11	1.04 ± 0.1	1.03 ± 0.08	NS	NS	NS
NPYSR‐Y2	1.03 ± 0.07	0.98 ± 0.08	0.87 ± 0.05	0.95 ± 0.05	NS	NS	NS
NPYSR‐Y4	1.06 ± 0.09	1.12 ± 0.12	0.89 ± 0.09	1.05 ± 0.11	NS	NS	NS
GR	1.27 ± 0.21	1.72 ± 0.32	1.94 ± 0.26	2.07 ± 0.29	*P *=* *0.07	NS	NS

The number of chicks used in each group was as follows: Saline CT 14; Saline HT 14; NPY CT 10 and NPY HT 14. Values are means ± SEM. NPY, neuropeptide Y; GR, Glucocorticoid receptor; NPYSRs, neuropeptide Y sub‐receptors ‐Y1, ‐Y2, and ‐Y4; CT, control thermoneutral treatment (30 ± 1°C); NS, Not significant.

NPYSRs have specific functions to carry out the stress response. For example, NPYSR‐Y1 mediates anxiolytic effect, whereas NPYSR‐Y2 mediates anxiogenic functions (Reichmann and Holzer 2016). In this study, it was found that the expression of NPYSRs‐Y5, ‐Y6, and ‐Y7 was stimulated by NPY, which indicates that the hypothermic functions of NPY were mediated through all or any of these receptors. NPY‐induced food intake in chickens occurred through NPYSRs‐Y1 and ‐Y5 (Holmberg et al. 2002), although NPYSR‐Y5 was also found to slightly contribute to food intake in mammals (Marsh et al. 1998). In this study, the mRNA expression of NPYSR‐Y1 was not changed, but that of NPYSR‐Y5 increased. Subsequently, it was further confirmed that a coinjection of CGP71683 (an NPYSR‐Y5 antagonist) plus NPY slightly attenuated the NPY‐induced hypothermia (Fig. 2D), which suggests that NPYSR‐Y5 is partially, but not entirely, involved with hypothermia. In addition, only NPYSR‐Y6 was increased by heat stress, suggesting that HT has a strong influence on NPYSR‐Y6. The function of NPYSRs‐Y6 and ‐Y7 in chickens remains unknown. However, until now, no antagonist has been available for NPYSRs‐Y6 and ‐Y7. Although it will be needed to analyze the protein level corresponding to the mRNA expression in future, previous reports (Boswell et al. 1998; He et al. 2016; Gao et al. 2017) showed that the mRNA expression of NPY and its receptors displayed the same pattern of changes with the protein expressions.

In this study, it was found that plasma concentrations of sodium, potassium, and chloride were not changed by NPY under CT or HT (*data not shown*), which indicates that NPY did not affect the electrolyte balance. This may have been due to the short experimental period (i.e., 60 min) and the specific feeding condition (i.e., fasting). It is possible that there might be an orexigenic effect of NPY under a longer experimental period with ad libitum feeding. Plasma glucose concentrations were lower in NPY‐treated chicks, and it could be speculated that the decline in glucose might be due to the anabolic function of NPY. Hypoglycemia is a well‐known phenomenon which combines with hypothermia in mammals (Buchanan et al. 1991) and amphibians (Branco 1997; Rocha and Branco 1998). Thaxton et al. (1974) reported that oral administration of glucose increased the body temperatures of the chicks that were exposed to a cold environment and suggested that carbohydrate metabolism is involved in the physiological regulation of body temperature. Moreover, Doerfler et al. (1998) found that hypoglycemia occurred in turkeys when hypothermia was also detected. Tachibana et al. (2006) reported that central administration of NPY reduced plasma glucose in fasted chicks, along with a reduction in body temperature, which is comparable with the current findings. Although it is not yet known how the decline in blood glucose causes a reduction in body temperature in birds, we can speculate that NPY might stimulate an energy‐consuming anabolic process to store glucose as glycogen in the liver and muscle and reduce catabolic processes of glycogen in the liver, and thereby reduce body heat so much as to cause hypothermia. Body temperature is very high in chickens (41.5°C) compared with humans (36.5–37.0°C), with the blood glucose level in chickens correspondingly very high (260 mg/100 mL), but low for humans (100 mg/100 mL), which indicates that there may be some relation between glucose level and body temperature since glucose can produce body heat. Kuenzel and McMurtry (1988) reported that central injection of NPY increased plasma insulin. Therefore, it could be possible that central NPY injection in this study increased peripheral insulin and reduced blood glucose and body heat.

In this study, we found that the plasma concentrations of histidine were decreased, but valine and tyrosine were increased, by heat stress. However, NPY did not show any effect that might modulate their levels. Amino acids have some roles in reducing body temperature in chicks (Erwan et al. 2014; Chowdhury et al. 2015, 2017). In rabbits, i.c.v. injections of taurine at 10 or 23°C caused hypothermia through reducing heat production and peripheral vasomotor tone to inhibit arousal level (Harris and Lipton 1977). Moreover, taurine increased in tissues (retina, nerves, kidney, and heart) to prevent leakage of the reactive compounds from the mitochondrial matrix, and thus indirectly acted as an antioxidant (Fang et al. 2002; Hansen et al. 2006). In addition, taurine improved lipid metabolism, reduced lipid peroxidation and increased growth performance in heat‐exposed chicks (Shim et al. 2006). In this study, we found that plasma taurine, a nonessential amino acid, increased as a result of central injection of NPY, which suggests that NPY might have stimulated the metabolic process to produce taurine in the tissue and to release it into the blood. We also found that i.c.v. injection of NPY increased the plasma anserine level. It has been reported that anserine decreased blood pressure and heart rate in rats (Tanida et al. 2010) and showed an antioxidant effect that prevented lipid peroxidation and scavenged free radicals in mammals (Kohen et al. 1988; Wu et al. 2003). Anserine, a dipeptide, is particularly abundant in the skeletal muscle and nervous tissue, and is found predominantly in birds (Aristoy et al. 2004). Therefore, it could be thought that an NPY‐mediated increase in plasma taurine and anserine may enhance physiological supports to enable chicks to adapt to heat stress.

Central administration of NPY has been found to stimulate the hypothalamic–pituitary–adrenal axis and increase the release of corticosterone in rats (Wahlestedt et al. 1987; Zarjevski et al. 1993; Small et al. 1997). In this study, it was also found that central injection of NPY increased the plasma corticosterone level under CT at 1 h; however, at the same time, the plasma corticosterone level decreased under heat stress. It could be speculated that NPY and heat stress might have stimulated to release more plasma corticosterone immediately after the exposure to heat stress, which might have caused GR‐mediated feedback regulation to reduce plasma corticosterone at 1 h. Moreover, we cannot preclude the possibility for being desensitized of NPY receptors somehow in the HT group. E is more potent than NE in causing vasoconstriction and increasing the heart rate, muscle strength, and blood pressure, as well as raising body temperature (Wurtman 2002). In this study, NPY caused a reduction in plasma E concentrations in HT chicks. Therefore, the reduced plasma corticosterone and E demonstrate the anti‐stress function of NPY in heat‐exposed fasted chicks.

In summary, central injection of NPY afforded thermotolerance along with increased mRNA expression of HSP‐70, ‐90, and NPYSRs (‐Y5, ‐Y6, and ‐Y7) in heat‐exposed chicks. The result of coinjection of NPY and CGP71683, an NPYSR‐Y5 antagonist, suggests that NPYSR‐Y5 may partially mediate the NPY‐induced hypothermia. Decreased levels of plasma glucose, corticosterone and E as well as increased plasma taurine and anserine further suggest that central NPY may serve to control thermal stress and body temperature to afford protective thermotolerance.

## Conflict of Interest

The authors declare that they have no conflicts of interest.

## References

[phy213511-bib-0001] Aksit, M. , S. Yalcın , S. Ozkan , K. Metin , and D. Ozdemir . 2006 Effects of temperature during rearing and crating on stress parameters and meat quality of broilers. Poult. Sci. 85:1867–1874. 17032815 10.1093/ps/85.11.1867

[phy213511-bib-0002] Aristoy, M. C. , C. Soler , and F. Toldrá . 2004 A simple, fast and reliable methodology for the analysis of histidine dipeptides as markers of the presence of animal origin proteins in feed for ruminants. Food Chem. 84:485–491.

[phy213511-bib-0003] Bahry, M. A. , V. S. Chowdhury , H. Yang , P. V. Tran , P. H. Do , G. Han , et al. 2017 Central administration of neuropeptide Y differentially regulates monoamines and corticosterone in heat‐exposed fed and fasted chicks. Neuropeptides 62:93–100. 27979380 10.1016/j.npep.2016.11.008

[phy213511-bib-0004] Boogers, I. , W. Pluggea , Y. Q. Stokkermansa , and B. Duchateau . 2008 Ultra‐performance liquid chromatographic analysis of amino acids in protein hydrolysates using an automated pre‐column derivatization method. J. Chromatogr. A 1189:406–409. 18070624 10.1016/j.chroma.2007.11.052

[phy213511-bib-0005] Boswell, T. , J. R. Millam , Q. Li , and I. C. Dunn . 1998 Cellular localization of neuropeptide Y mRNA and peptide in the brain of the Japanese quail and domestic chicken. Cell Tissue Res. 293:31–38. 9634595 10.1007/s004410051095

[phy213511-bib-0006] Boswell, T. , I. C. Dunn , and S. A. Corr . 1999 Hypothalamic neuropeptide Y mRNA is increase after feed restriction in growing broilers. Poult. Sci. 78:1203–1207. 10472848 10.1093/ps/78.8.1203

[phy213511-bib-0007] Branco, L. G. S. 1997 Effects of 2‐deoxy‐D‐glucose and insulin on plasma glucose levels and behavioral thermoregulation of toads. Am. J. Physiol. 272:R1–R5. 9038984 10.1152/ajpregu.1997.272.1.R1

[phy213511-bib-0008] Bromée, T. , P. Sjödin , R. Fredriksson , T. Boswell , T. A. Larsson , E. Salaneck , et al. 2006 Neuropeptide Y‐family receptors Y6 and Y7 in chicken. Cloning, pharmacological characterization, tissue distribution and conserved synteny with human chromosome region. FEBS J. 273:2048–2063. 16640567 10.1111/j.1742-4658.2006.05221.x

[phy213511-bib-0009] Buchanan, T. A. , P. Cane , C. C. Eng , G. F. Sipos , and C. Lee . 1991 Hypothermia is critical for survival during prolonged insulin‐induced hypoglycemia in rats. Metabolism 40:330–334. 2000047 10.1016/0026-0495(91)90118-g

[phy213511-bib-0010] Chen, X. , Y. Zhu , X. Cheng , Z. Zhang , and S. Xu . 2012 The protection of selenium against cadmium‐induced cytotoxicity via the heat shock protein pathway in chicken splenic lymphocytes. Molecules 17:14565–14572. 23222903 10.3390/molecules171214565 PMC6268861

[phy213511-bib-0011] Chowdhury, V. S. , S. Tomonaga , S. Nishimura , S. Tabata , and M. Furuse . 2012 Physiological and behavioral responses of young chicks to high ambient temperature. J. Poult. Sci. 49:212–218.

[phy213511-bib-0012] Chowdhury, V. S. , S. Tomonaga , T. Ikegami , E. Erwan , K. Ito , J. F. Cockrem , et al. 2014 Oxidative damage and brain concentrations of free amino acid in chicks exposed to high ambient temperature. Comp. Biochem. Physiol. A: Mol. Integr. Physiol. 169:70–76. 24389089 10.1016/j.cbpa.2013.12.020

[phy213511-bib-0013] Chowdhury, V. S. , A. Shigemura , E. Erwan , K. Ito , M. A. Bahry , P. V. Tran , et al. 2015 Oral administration of L‐citrulline, but not L‐arginine or L‐ornithine, acts as a hypothermic agent in chicks. J. Poult. Sci. 52:331–335.

[phy213511-bib-0014] Chowdhury, V. S. , G. Han , M. A. Bahry , P. V. Tran , P. H. Do , H. Yang , et al. 2017 L‐Citrulline acts as potential hypothermic agent to afford thermotolerance in chicks. J. Therm. Biol 69:163–170. 29037378 10.1016/j.jtherbio.2017.07.007

[phy213511-bib-0015] Cline, M. A. , and M. Furuse . 2012 Neuropeptide regulation of food intake in chicks Pp 1–34 in Morrison J. L., ed. Food intake: regulation, assessing and controlling. NOVA Science Publishers, Inc., Hauppauge New York.

[phy213511-bib-0016] Dark, J. , and K. M. Pelz . 2008 NPY Y1 receptor antagonist prevents NPY‐induced torpor like hypothermia in cold‐acclimated Siberian hamsters. Am. J. Physiol. Regul. Integr. Comp. Physiol. 94:R236–R245. 10.1152/ajpregu.00587.2007 17989140

[phy213511-bib-0017] Davis, J. L. , D. T. Masuoka , L. K. Gerbrandt , and A. Cherkin . 1979 Autoradiographic distribution of L‐proline in chicks after intracerebral injection. Physiol. Behav. 22:693–695. 482410 10.1016/0031-9384(79)90233-6

[phy213511-bib-0018] Denbow, D. M. , and M. A. Cline . 2015 Food intake regulation Pp. 469–485 in Scanes C. G., ed. Sturkie's Avian Physiology. Academic Press, New York.

[phy213511-bib-0019] Doerfler, R. E. , F. W. Edens , C. R. Parkhurst , G. B. Havenstein , and M. A. Qureshi . 1998 Hypothermia, hypoglycemia, and hypothyrosis associated with poult enteritis and mortality syndrome. Poult. Sci. 77:1103–1109. 9706073 10.1093/ps/77.8.1103 PMC7107120

[phy213511-bib-0020] Ebeid, T. A. , T. Suzuki , and T. Sugiyama . 2012 High temperature influences eggshell quality and calbindin‐D28k localization of eggshell gland and all intestinal segments of laying hens. Poult. Sci. 91:2282–2287. 22912464 10.3382/ps.2011-01898

[phy213511-bib-0021] Erwan, E. , V. S. Chowdhury , M. Nagasawa , R. Goda , T. Otsuka , S. Yasuo , et al. 2014 Oral administration of D‐aspartate, but not L‐aspartate, depresses rectal temperature and alters plasma metabolites in chicks. Life Sci. 109:65–71. 24881518 10.1016/j.lfs.2014.05.015

[phy213511-bib-0022] Fang, Y. Z. , S. Yang , and G. Wu . 2002 Free radicals, antioxidants, and nutrition. Nutrition 18:872–879. 12361782 10.1016/s0899-9007(02)00916-4

[phy213511-bib-0023] Fasulo, S. , S. Marino , A. Mauceri , M. Maisano , A. Giannetto , A. D'Agata , et al. 2010 A multibiomarker approach in Coris julis living in a natural environment. Ecotoxicol. Environ. Saf. 73:1565–1573. 20132985 10.1016/j.ecoenv.2010.01.008

[phy213511-bib-0024] Feder, M. E. , and G. E. Hofmann . 1999 Heat‐shock proteins, molecular chaperones, and the stress response: evolutionary and ecological physiology. Annu. Rev. Physiol. 61:243–282. 10099689 10.1146/annurev.physiol.61.1.243

[phy213511-bib-0025] Furuse, M. , M. Matsumoto , N. Saito , K. Sugahara , and S. Hasegawa . 1997 The central corticotropin‐releasing factor and glucagon‐like peptide‐1 in food intake of the neonatal chick. Eur. J. Pharmacol. 339:211–214. 9473137 10.1016/s0014-2999(97)01391-5

[phy213511-bib-0026] Furuse, M. , R. Ando , T. Bungo , R. Ao , M. Shimojo , and Y. Masuda . 1999 Intracerebroventricular injection of orexins does not stimulate food intake in neonatal chicks. Br. Poult. Sci. 40:698–700. 10670685 10.1080/00071669987115

[phy213511-bib-0027] Gao, S. , J. Zhang , C. He , F. Meng , G. Bu , G. Zhu , et al. 2017 Molecular characterization of neuropeptide Y (NPY) receptors (Y1, Y4 and Y6) and investigation of the tissue expression of their ligands (NPY, PYY and PP) in chickens. Gen. Comp. Endocrinol. 240:46–60. 27641685 10.1016/j.ygcen.2016.09.005

[phy213511-bib-0028] Hansen, S. H. , M. L. Andersen , H. Birkedal , C. Cornett , and F. Wibrand . 2006 The importance role of taurine in oxidative metabolism. Adv. Exp. Med. Biol. 58:129–135. 10.1007/978-0-387-33504-9_13 17153596

[phy213511-bib-0029] Hao, Y. , and X. H. Gu . 2014 Effects of heat shock protein 90 expression on pectoralis major oxidation in broilers exposed to acute heat stress. Poult. Sci. 1:2709–2717. 10.3382/ps.2014-03993 25239533

[phy213511-bib-0030] Harris, W. S. , and J. M. Lipton . 1977 Intracerebroventricular taurine in rabbits: effects on normal body temperature, endotoxin fever and hyperthermia produced by PGE1 and amphetamine. J. Physiol. 266:397–410. 857004 10.1113/jphysiol.1977.sp011773 PMC1283571

[phy213511-bib-0031] He, C. , J. Zhang , S. Gao , F. Meng , G. Bu , G. Li , et al. 2016 Molecular characterization of three NPY receptors (Y2, Y5 and Y7) in chickens: Gene structure, tissue expression, promoter identification, and functional analysis. Gen. Comp. Endocrinol. 236:24–34. 27142335 10.1016/j.ygcen.2016.04.019

[phy213511-bib-0032] Holmberg, S. K. S. , S. Mikko , T. Boswell , R. Zoorob , and D. Larhammar . 2002 Pharmacological characterization of cloned chicken neuropeptide Y receptors Y1 and Y5. J. Neurochem. 81:462–471. 12065655 10.1046/j.1471-4159.2002.00817.x

[phy213511-bib-0033] Ito, K. , E. Erwan , M. Nagasawa , M. Furuse , and V. S. Chowdhury . 2014 Changes in free amino acid concentrations in the blood, brain and muscle of heat‐exposed chicks. Br. Poult. Sci. 55:644–652. 25157850 10.1080/00071668.2014.957653

[phy213511-bib-0034] Ito, K. , M. A. Bahry , Y. Hui , M. Furuse , and V. S. Chowdhury . 2015 Acute heat stress up‐regulates neuropeptide Y precursor mRNA expression and alters brain and plasma concentrations of free amino acids in chicks. Comp. Biochem. Physiol. A: Mol. Integr. Physiol. 187:13–19. 25933935 10.1016/j.cbpa.2015.04.010

[phy213511-bib-0035] Jakob, U. , H. Lilie , I. Meyer , and J. Buchner . 1995 Transient interaction of Hsp90 with early unfolding intermediates of citrate synthase. Implications for heat shock in vivo. J. Biol. Chem. 270:7288–7294. 7706269 10.1074/jbc.270.13.7288

[phy213511-bib-0036] Kobayashi, K. , and K. S. Pillai (eds). 2013 Transformation of data and outliers Pp. 37–46 in A Handbook of Applied Statistics in Pharmacology. CRC Press: Taylor & Francis Group, New York.

[phy213511-bib-0037] Kohen, R. , Y. Yamamoto , C. K. Cundy , and N. B. Ames . 1988 Antioxidant activity of carnosine, homocarnosine and anserine present in muscle and brain. Proc. Natl Acad. Sci. USA 85:3175–3179. 3362866 10.1073/pnas.85.9.3175 PMC280166

[phy213511-bib-0038] Kuenzel, W. J. , and M. Masson . 1988 A Stereotaxic Atlas of the Brain of the Chick (Gallus domesticus). The Johns Hopkins University Press, Baltimore.

[phy213511-bib-0039] Kuenzel, W. J. , and J. McMurtry . 1988 Neuropeptide Y: brain localization and central effects on plasma insulin levels in chicks. Physiol. Behav. 44:669–678. 3070587 10.1016/0031-9384(88)90334-4

[phy213511-bib-0040] Kuenzel, W. J. , L. W. Douglass , and B. A. Davison . 1987 Robust feeding following central administration of neuropeptide Y or peptide YY in chicks. Gallus domesticus. Peptides 8:823–828. 3432131 10.1016/0196-9781(87)90066-0

[phy213511-bib-0041] Larsson, T. A. , F. Olsson , G. Sundström , L. G. Lundin , S. Brenner , B. Venkatesh , et al. 2008 Early vertebrate chromosome duplications and the evolution of the neuropeptide Y receptor gene regions. BMC Evol. Biol. 8:184. 18578868 10.1186/1471-2148-8-184 PMC2453138

[phy213511-bib-0042] Lundell, I. , T. Boswell , and D. Larhammar . 2002 Chicken neuropeptide Y‐family receptor Y4: a receptor with equal affinity for pancreatic polypeptide, neuropeptide Y and peptide YY. J. Mol. Endocrinol. 28:225–235. 12063188 10.1677/jme.0.0280225

[phy213511-bib-0043] Malva, J. O. , S. Xapelli , S. Baptista , J. Valero , F. Agasse , R. Ferreira , et al. 2012 Multifaces of neuropeptide Y in the brain ‐ neuroprotection, neurogenesis and neuroinflammation. Neuropeptides 46:299–308. 23116540 10.1016/j.npep.2012.09.001

[phy213511-bib-0044] Marsh, D. J. , G. Hollopeter , K. E. Kafer , and R. D. Palmiter . 1998 Role of the Y5 neuropeptide Y receptor in feeding and obesity. Nat. Med. 4:718–721. 9623983 10.1038/nm0698-718

[phy213511-bib-0045] Miyata, Y. , and I. Yahara . 1992 The 90‐kDa heat shock protein, Hsp90 binds and protects casein kinase II from self‐aggregation and enhances its kinase activity. J. Biol. Chem. 267:7042–7045. 1551911

[phy213511-bib-0046] Najafi, P. , I. Zulkifli , N. A. Jajuli , A. S. Farjam , S. K. Ramiah , A. A. Amir , et al. 2015 Environmental temperature and stocking density effects on acute phase proteins, heat shock protein 70, circulating corticosterone and performance in broiler chickens. Int. J. Biometeorol. 59:1577–1583. 25649005 10.1007/s00484-015-0964-3

[phy213511-bib-0047] Nollen, E. A. A. , and R. I. Morimoto . 2002 Chaperoning signaling pathways: molecular chaperones as stress‐sensing “heat shock” proteins. J. Cell Sci. 115:2809–2816. 12082142 10.1242/jcs.115.14.2809

[phy213511-bib-0048] Persaud, S. J. , and G. A. Bewick . 2014 Peptide YY: more than just an appetite regulator. Diabetologia 57:1762–1769. 24917132 10.1007/s00125-014-3292-y

[phy213511-bib-0049] Pockley, A. G. 2003 Heat shock proteins as regulators of the immune response. Lancet 362:469–476. 12927437 10.1016/S0140-6736(03)14075-5

[phy213511-bib-0050] Quinteiro‐Filho, W. M. , A. Ribeiro , V. Ferraz‐de‐Paula , M. L. Pinheiro , M. Sakai , L. R. M. Sá , et al. 2010 Heat stress impairs performance parameters induces intestinal injury and decreases macrophage activity in broiler chickens. Poult. Sci. 89:1905–1914. 20709975 10.3382/ps.2010-00812

[phy213511-bib-0051] Reichmann, F. , and P. Holzer . 2016 Neuropeptide Y: a stressful review. Neuropeptides 55:99–109. 26441327 10.1016/j.npep.2015.09.008 PMC4830398

[phy213511-bib-0052] Richter, K. , M. Haslbeck , and J. Buchner . 2010 The heat shock response: life on the verge of death. Mol. Cell 40:253–266. 20965420 10.1016/j.molcel.2010.10.006

[phy213511-bib-0053] Rocha, P. L. , and L. G. S. Branco . 1998 Physiological significance of behavioral hypothermia in hypoglycemic frogs (*Rana catesbeiana*). Comp. Biochem. Physiol. A: Mol. Integr. Physiol. 19:957–961. 10.1016/s1095-6433(98)00010-5 9773488

[phy213511-bib-0054] Rozenboim, I. , E. Tako , O. Gal‐Garber , J. A. Proudman , and Z. Uni . 2007 The effect of heat stress on ovarian function of laying hens. Poult. Sci. 86:1760–1765. 17626822 10.1093/ps/86.8.1760

[phy213511-bib-0055] Saito, E. S. , H. Kaiya , T. Tachibana , S. Tomonaga , D. M. Denbow , K. Kangawa , et al. 2005 Inhibitory effect of ghrelin on food intake is mediated by the corticotropin releasing factor system in neonatal chicks. Regul. Pept. 125:201–208. 15582733 10.1016/j.regpep.2004.09.003

[phy213511-bib-0056] Sandercock, D. A. , R. R. Hunter , G. R. Nute , M. A. Mitchell , and P. M. Hocking . 2001 Acute heat stress‐induced alterations in blood acid‐base status and skeletal muscle membrane integrity in broiler chickens at two ages: implications for meat quality. Poult. Sci. 80:418–425. 11297279 10.1093/ps/80.4.418

[phy213511-bib-0057] Schmittgen, T. D. , and K. J. Livak . 2008 Analyzing real‐time PCR data by the comparative (CT) method. Nat. Protoc. 3:1101–1108. 18546601 10.1038/nprot.2008.73

[phy213511-bib-0058] Shim, K. S. , K. T. Hwang , M. W. Son , and G. H. Park . 2006 Lipid metabolism and peroxidation in broiler chicks under chronic heat stress. Asian‐Australas J. Anim. Sci. 19:1206–1211.

[phy213511-bib-0059] Small, C. J. , D. G. A. Morgan , K. Meeran , M. M. Heath , I. Gunn , C. M. B. Edwards , et al. 1997 Peptide analogue studies of the hypothalamic neuropeptide Y receptor mediating pituitary adrenocorticotrophic. Proc. Natl Acad. Sci. USA 94:11686–11691. 9326671 10.1073/pnas.94.21.11686 PMC23590

[phy213511-bib-0060] Stetler, R. A. , Y. Gan , W. Zhang , A. K. Liou , Y. Gao , G. Cao , et al. 2010 Heat shock proteins: cellular and molecular mechanisms in the central nervous system. Prog. Neuropsychopharmacol. Biol. 92:184–211. 10.1016/j.pneurobio.2010.05.002 PMC2939168 20685377

[phy213511-bib-0061] Szekely, M. , E. Petervari , and Z. Szelenyi . 2004 Orexigenic vs. anorexigenic peptides and feeding status in the modulation of fever and hypothermia. Front Biosci. 9:2746–2763. 15353311 10.2741/1433

[phy213511-bib-0062] Tachibana, T. , S. Saito , S. Tomonaga , T. Takagi , E. S. Saito , T. Nakanishi , et al. 2004 Effect of central administration of prolactin‐releasing peptide on feeding in chicks. Physiol. Behav. 80:713–719. 14984806 10.1016/j.physbeh.2003.12.005

[phy213511-bib-0063] Tachibana, T. , M. Sato , D. Oikawa , H. Takahashi , T. Boswell , and M. Furuse . 2006 Intracerebroventricular injection of neuropeptide Y modifies carbohydrate and lipid metabolism in chicks. Regul. Pept. 136:1–8. 16713643 10.1016/j.regpep.2006.04.005

[phy213511-bib-0064] Tachibana, T. , D. Oikawa , N. Adachi , T. Boswell , and M. Furuse . 2007 Central administration of alpha‐melanocyte‐stimulating hormone changes lipid metabolism in chicks. Comp. Biochem. Physiol. A: Mol. Integr. Physiol. 148:408–412. 17600745 10.1016/j.cbpa.2007.05.023

[phy213511-bib-0065] Takahashi, H. , M. Iigo , K. Ando , T. Tachibana , D. M. Denbow , and M. Furuse . 2005 Regulation of body temperature by thyrotropin‐releasing hormone in neonatal chicks. Dev. Brain Res. 157:58–64. 15939085 10.1016/j.devbrainres.2005.03.004

[phy213511-bib-0066] Tanida, M. , J. Shen , D. Kubomura , and K. Nagai . 2010 Effects of anserine on the renal sympathetic nerve activity and blood pressure in urethane‐anesthetized rats. Physiol. Res. 59:177–185. 19537934 10.33549/physiolres.931623

[phy213511-bib-0067] Thaxton, P. , R. D. Wyatt , and P. B. Hamilton . 1974 The effect of environmental temperature on paratyphoid infection in the neonatal chicken. Poult. Sci. 53:88–94. 4208104 10.3382/ps.0530088

[phy213511-bib-0068] Tu, W. L. , C. Y. Cheng , S. H. Wang , P. C. Tang , C. F. Chen , H. H. Chen , et al. 2016 Profiling of differential gene expression in the hypothalamus of broiler‐type Taiwan country chickens in response to acute heat stress. Theriogenology 85:483–494. 26462659 10.1016/j.theriogenology.2015.09.028

[phy213511-bib-0069] Wahlestedt, C. , G. Skagerberg , R. Ekman , M. Heilig , F. Sundler , and R. Hhkanson . 1987 Neuropeptide Y (NPY) in the area of the hypothalamic paraventricular nucleus activates the pituitary‐adrenocortical axis in the rat. Brain Res. 417:33–38. 3040184 10.1016/0006-8993(87)90176-4

[phy213511-bib-0070] Wu, H. , C. Shiau , H. Chen , and T. Chiou . 2003 Antioxidant activities of carnosine, anserine, some free amino acids and their combination. J. Food Drug Anal. 11:148–153.

[phy213511-bib-0071] Wurtman, R. J. 2002 Stress and the adrenocortical control of epinephrine synthesis. Metabolism 51:11–14. 12040535 10.1053/meta.2002.33185

[phy213511-bib-0072] Yi, J. , E. R. Gilbert , P. B. Siegel , and M. A. Cline . 2015 Fed and fasted chicks from lines divergently selected for low or high body weight have differential hypothalamic appetite‐associated factor mRNA expression profiles. Behav. Brain Res. 286:58–63. 25677648 10.1016/j.bbr.2015.02.008

[phy213511-bib-0073] Zarjevski, N. , I. Cusin , R. Vettor , F. Rohner‐Jeanrenaud , and B. Jeanrenaud . 1993 Chronic intracerebroventricular neuropeptide‐Y administration to normal rats mimics hormonal and metabolic changes of obesity. Endocrinology 133:1753–1758. 8404618 10.1210/endo.133.4.8404618

[phy213511-bib-0074] Zhao, L. , and W. A. Jones . 2012 Expression of heat shock protein genes in insect stress responses. Invertebrate Surviv. J. 9:93–101.

